# Extended multi-organ resection for cT4 gastric carcinoma: A retrospective analysis

**DOI:** 10.12669/pjms.292.2898

**Published:** 2013-04

**Authors:** Longbin Xiao, Mingzhe Li, Fengfeng Xu, Huishao Ye, Wenhui Wu, Shuo Long, Wenfeng Li, Yulong He

**Affiliations:** 1 Longbin Xiao, Department of Gastrointestinal and Pancreatic Surgery, The First Affiliated Hospital, Sun Yat-sen University, Guangzhou, China, 510700.; 2 Mingzhe Li, Department of Gastrointestinal and Pancreatic Surgery, The First Affiliated Hospital, Sun Yat-sen University, Guangzhou, China, 510700.; 3 Fengfeng Xu, Department of General Surgery I, Huangpu Division of the First Affiliated Hospital of Sun Yat-sen University, Guangzhou, China, 510700.; 4 Huishao Ye, Department of Pharmacy, Huangpu Division of the First Affiliated Hospital of Sun Yat-sen University, Guangzhou, China, 510700.; 5 Wenhui Wu, Department of Gastrointestinal and Pancreatic Surgery, The First Affiliated Hospital, Sun Yat-sen University, Guangzhou, China, 510700.; 6 Shuo Long, Department of Gastrointestinal and Pancreatic Surgery, The First Affiliated Hospital, Sun Yat-sen University, Guangzhou, China, 510700.; 7 Wenfeng Li, Department of Gastrointestinal and Pancreatic Surgery, The First Affiliated Hospital, Sun Yat-sen University, Guangzhou, China, 510700.; 8 Yulong He, Department of Gastrointestinal and Pancreatic Surgery, The First Affiliated Hospital, Sun Yat-sen University, Guangzhou, China, 510700.

**Keywords:** Gastric cancer, Prognostic factors, Multiorgan resection

## Abstract

***Objective: ***Combined resection for locally advanced (T4) gastric cancer may result in high morbidity and mortality. The aim of this study was to evaluate the clinicopathologic characteristics to determine the prognostic factors for T4 gastric cancers.

***Methodology:*** A total of 463 consecutive patients with gastric cancers were enrolled in this study. Among them, 63 patients received combined resections. Various clinicopathologic factors influencing survival rates were evaluated. Survival rates were calculated according to the Kaplan-Meier method. Prognostic factors were evaluated by the univariate and multivariate analysis.

***Results:*** Thirty-one patients (49.2%) received one additional organ resection and 32 patients (50.8%) received two or more additional organ resections. Curative resection was performed in 49 patients (77.8%). Multivariate analysis identified curative resection (hazard ratio 0.330; 95 percent confidence interval, 0.139-0.784; *P* = 0.012) and tumor diameter (> 7 cm) (hazard ratio, 3.589; the 95 percent confidence interval, 1.425-9.037; *P* = 0.007) as independent prognostic factor for patients with T4 gastric cancer undergoing combined resection.

***Conclusions: ***The use of aggressive multi-organ resection was recommended for patients with T4 gastric carcinoma, with tumor diameter as a useful indicator. Patients with relatively small tumor diameter (≤ 7cm) could benefit from multi-organ resections.

## INTRODUCTION

The overall 5-year survival rate for locally advanced (T4) gastric carcinoma patients is generally below 20%.^1^ Radical resection is considered the best approach for achieving better long-term survival for patients.^2^^-^^4^ For the locally advanced (T4) gastric cancer with adjacent organ infiltration, multi-organ resection is required to achieve curative resection (R0) with negative macroscopic and microscopic margins. However, the combined multi-organ resection has an associated high risk of morbidity and mortality.^5^ Aggressive surgical treatment and appropriate patient selection for the locally advanced gastric cancer remain controversial. And few studies have demonstrated the clinicopathologic characteristics and the prognostic factors for T4 gastric cancers.

In this study, multi-organ resection was defined as the simultaneous resection of the stomach and directly invaded organs. The curative (R0) resection was defined as the complete removal of cancer tissue with no residual tumor macroscopically or microscopically and no evidence of distant metastasis. The surgery date was considered to be the entry point. The aim of this retrospective study was to study the clinicopathologic characteristics in patients with T4 gastric cancers. We have also investigated the prognostic factors of survival, which may serve as indicators of what types of patients who should receive and benefit from multi-organ resection.

## METHODOLOGY

Four hundred sixty three (463) consecutive patients with gastric cancers were enrolled in this study. Prior written and informed consent was obtained from every patient and the study was approved by the ethics review board of the first affiliated hospital, Sun Yat-sen University. Among these patients, patients who were diagnosed with clinical T4 gastric carcinoma (cT4) that invaded adjacent organs, and underwent extended multiorgan resection were analyzed retrospectively. Diagnosis of T4 disease was based on clinical data, including computed tomography (CT) scan data, endoscopic ultrasonography (US), and intraoperative assessments.

Multi-organ resection was defined as the simultaneous resection of the stomach and directly invaded organs. The curative (R0) resection was defined as the complete removal of cancer tissue with no residual tumor macroscopically or microscopically and no evidence of distant metastasis. The surgery date was considered to be the entry point. The date of dead, or the most recent follow-up time if the patients were not dead, was defined as the end point of the study. The following patients were excluded: (i): patients who were lost to follow-up or without complete clinical data; (ii) patients who had undergone resection of the distal esophagus for proximal expansion of the tumor, splenectomy for lymph node dissection, or additional procedures owing to disorders other than tumor invasion.

Data including clinicopathologic features of patients, such as ages, gender, tumor locations, tumor diameters, gastrectomy types, Borrmann types, histologic types, depths of invasion, lymph node stage and TNM stage, were obtained from a prospectively recorded database and given in Table-I. Histological classification and staging were based on the 6^th^ edition of the UICC TNM classification.^6^

***Statistical analyses: ***Statistic analysis was finished by The SPSS program version 13.0 for Windows (SPSS Inc, Chicago, IL, USA). The outcome of interest was the prognostic factors for T4 gastric cancers. All categorical data were presented as rate and continuous data were expressed as mean ± SD. The strata were defined by the clinicopathologic variables of interest, including ages, gender, tumor locations, tumor diameters, gastrectomy types, Borrmann types, histologic types, depth of invasion, lymph node stage and TNM stage. The Kaplan-Meier method was used to define survival-associated factors. Differences in survival were analyzed using the Log rank tests. All factors associated with survival with a probability value of 0.05 or less were selected for multivariate analysis. The Cox proportional hazards model (Cox’s model) was used for multivariate analysis to define prognostic factors determining cancer related survival. Hazard ratios (HR) with 95% CI were obtained as a measurement of association. *P *value less than 0.05 was considered statistically significant.

## RESULTS

***Clinicopathologic characteristics: ***Of the 463 patients with gastric cancers enrolled in this study, 64 patients were diagnosed with clinical T4 gastric carcinoma (cT4) that invaded adjacent organs, and underwent extended multi-organ resections. One patient, who was lost to follow-up, was excluded. Therefore, a total of 63 cases were included in the analysis.

The clinicopathologic characteristics of the 63 patients are given in Table-I. Their mean age was 56.6 ± 12.6 years, ranging between 35 and 84 years. These patients comprised 40 (63.5%) male and 23 (36.5%) female. The mean tumor diameter was 7.2 ± 4.2 cm. Sixteen patients (25.4%) underwent distal subtotal gastrectomy and 47 (74.6%) underwent total gastrectomy. The most common lesion site was the upper third (38.1%), and the most common type was Borrmann III (49.2%). Histopathologic examination confirmed pT4 disease in 25 patients (39.7%), while 38 (60.3%) patients were pT3. The most common pathological type was poor-undifferentiated (76.2%). Fifty-one (81.0%) patients had lymph node metastasis. Among them, 19 (30.2%) had pN1, 15 (23.8%) had pN2, and 17 (27.0%) had pN3 disease. Six (9.5%) patients were in stage II; 21 (33.3%) in stage III; and 36 (57.1%) in stage IV, according to the TNM classification. Thirty-one patients (49.2%) underwent single-organ resection, and 32 patients (50.8%) had two or more organs resected. Curative resection was carried out in 49 patients (77.8%) and noncurative resection performed in 14 patients (22.2%).

**Fig.1:**
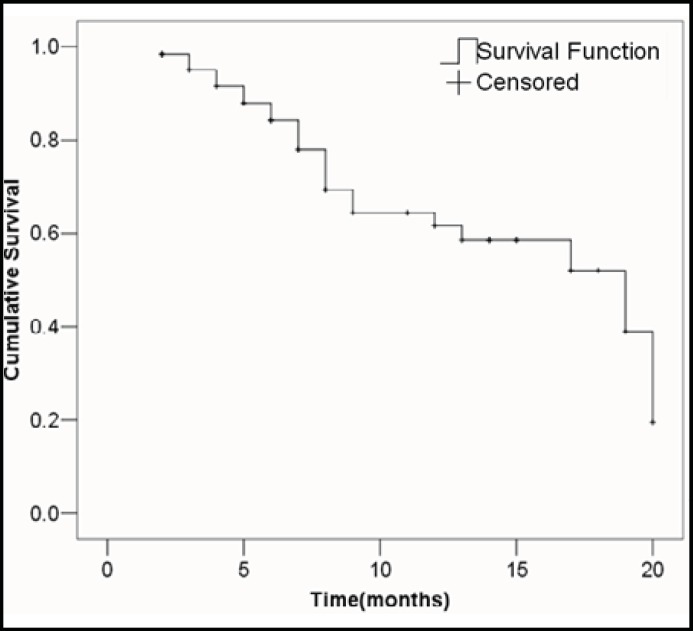
Cumulative survival rates of patients with T4 gastric cancers.

The median follow-up time was 13 months (range, 1-22 months). Kaplan-Meier analysis of the cumulative survival rate was 61.6% at one year after operation (Fig.1). The median for survival time was 19.0 months. The 95 percent confidence interval (CI) is 11.08-26.91. Standard D2 lymph node dissections or D2 plus para-aortic lymph node dissections were performed in most surgeries with curative intent.

**Fig.2:**
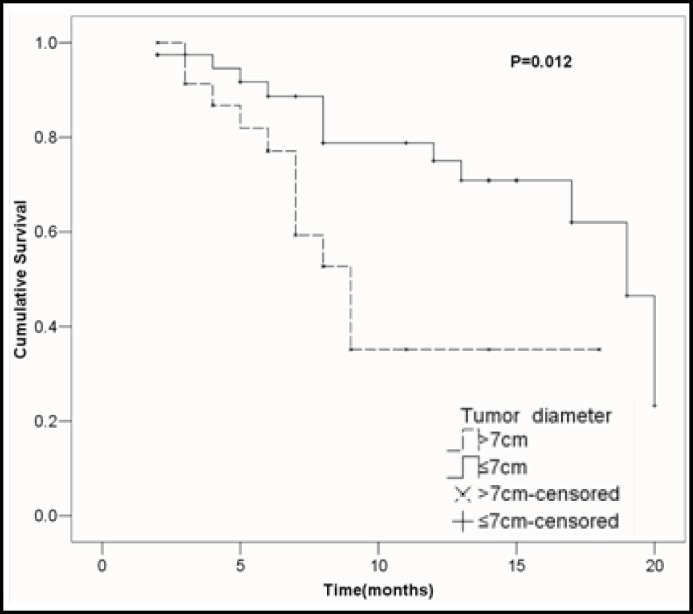
Cumulative survival rates in relation to tumor diameters, together with the *P *value from the log-rank tests.

***Univariate analysis of clinicopathologic variables: ***The effects of clinicopathologic variables on the overall survival rate were analyzed using the Kaplan-Meier method, and the results of the log-rank tests for each variable are given in Table-II. To identify the putative clinical variables that may be able to be used as indicators of survival rate, univariate analysis was performed. Univariate analysis identified two clinical variables associated with survival rate: tumor diameter (1-year survival rate: 75.0% among patients with tumor diameter≤7cm and 35.1% among patients with tumor diameter＞7cm; P =0 .012; Fig.2), and curability (1-year survival rate: 68.2% among patients with curative resection and 41.9% among patients with noncurative resection; *P *=0 .030; Fig.3).

***Multivariable analysis:*** Table-III shows the results of the final multivariable Cox proportional hazards model with only tumor diameter and curability. In this model, tumor diameter > 7cm (HR, 3.589; 95% CI, 1.425-9.037; *P* = 0.007) was significantly associated with a lower survival rate, while curative resection (HR, 0.330; 95% CI, 0.139-0.784; *P* = .0.012) was significantly associated with a higher survival rate.

## DISCUSSION

Gastric cancer remains second leading cause of death in the world, accounting for 10% of newly diagnosed cancers.^7^^,^^8^ Complete resection is the only potentially curative treatment. However, long-term survival after gastrectomy for locally advanced (T4) gastric cancer remains poor.^9^ Because of the associated high risk of postoperative morbidity and mortality, and the poor survival rate, radical gastrectomy with multiorgan resection is still a controversial question for patients with gastric cancer invading adjacent organs. Thus, the decision of whether or not to perform radical gastrectomy with multiorgan resection of invaded organs must be decided by the experienced surgeon and considering some prognostic factors.

**Table-I:** Clinicopathologic characteristics of 63 Patients

*Clinical variable*	*Mean or patients*	*SD or rate*
*Age *(y)	56.6	12.6
*Gender *		
Male	40	63.5%
Female	23	36.5%
*Tumor diameter *(cm)	7.2	4.2
*Tumor location*		
Upper	24	38.1%
Middle	17	27.0%
Lower	15	23.8%
Whole	7	11.1%
*Operation type*		
Subtotal gastrectomy	16	25.4%
Total gastrectomy	47	74.6%
*Borrmann type*		
I and II	10	15.9%
III	31	49.2%
IV	22	34.9%
*Differentiation*		
Well-moderate	15	23.8%
Poor-undifferentiated	48	76.2%
*Depth of invasion*		
T3	38	60.3%
T4	25	39.7%
*N-stage*		
N0	12	19.0%
N1(1~6)	19	30.2%
N2(7~15)	15	23.8%
N3(≥16)	17	27.0%
*TNM stage*		
II	6	9.5%
III	21	33.3%
IV	36	57.1%
*Number of organs resected*		
1	31	49.2%
≥2	32	50.8%
*Curability*		
Non-curative	14	22.2%
Curative	49	77.8%

**Table-II:** Univariate analysis of potential prognostic factors for survival rate in patients with T4 gastric cancer

*Variables*	*1-yr survival rate (%)*	*Log-rank P*
*Age*(y)		
＜65	55.0	0.799
≥65	73.5	
*Gender*		
Male	85.8	0.731
Female	64.6	
Tumor diameter (cm)*		
≤7	75.0	0.012
＞7	35.1	
*Operation type*		
Subtotal gastrectomy	77.9	0.579
Total gastrectomy	56.0	
*Borrmann type*		
I and II	76.2	0.894
III	84.6	
IV	63.9	
*Differentiation*		
Well-moderate	91.7	0.103
Poor-undifferentiated	52.9	
*Depth of invasion*		
T3	62.8	0.373
T4	60.5	
*N-stage*		
N0	80.0	0.263
N1(1~6)	80.7	
N2(7~15)	45.7	
N3(≥16)	46.9	
*TNM stage*		
II	100	0.105
III	60.6	
IV	56.3	
*Number of organs resected*		
1	66.3	0.241
≥2	56.6	
Curability*		
Non-curative	41.9	0.030
Curative	68.2	

*P value <0.05

**Figure:**
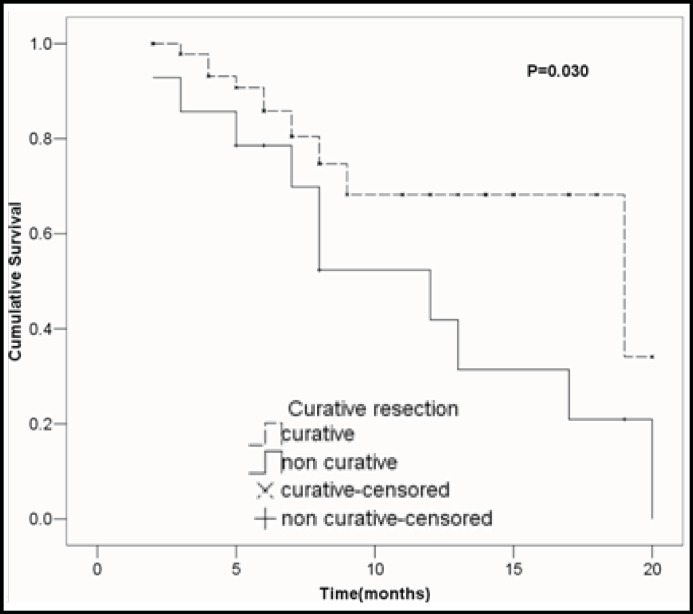
**Fig.3** **: **Cumulative survival rates in relation to curability, together with the *P *value from the log-rank tests.

**Table-III:** Multivariable Cox regression analysis of prognostic factors associated with survival time in patients with T4 gastric cancer

*Clinical variable*	*Model coefficient*	*HR*	*95% CI for HR*	*P value*
*Tumor diameter (cm)*				
≤7		1.000		
>7	1.278	3.589	1.425-9.037	0.007
*Curability*				
Non-curative		1.000		
Curative	-1.110	0.330	0.139-0.784	0.012

Note: HR, hazard ratio

In the current study, we presented many clinicopathologic variables of T4 gastric cancer, such as ages, gender, tumor locations, tumor diameters, gastrectomy types, Borrmann types, histologic types, depth of invasion, lymph node stage and TNM stage. The clinicopathologic characteristics of T4 gastric cancer in our patients were similar to those as reported previously.^10^^,^^11^ Total gastrectomy was most common surgical procedure (74.6%). The most common pathological type was poor-undifferentiated (approximately, 77%) and more than 80% patients had lymph node metastasis.

We observed that only 25 patients (39.7%) were confirmed with pT4 disease by histopathologic examinations, while more than half of the patients were with pT3 disease. It could be difficult to correctly identify the T4 gastric cancer by computed tomography scan, endoscopic ultrasonography, and intraoperative assessments. Because many pT3 patients showed densely inflammatory adhesions between the cancer and adjacent organs without actual invasion, the positive predictive values of preoperative computed tomographic scans in assessing T4 stage were only 50%.^12^ In the current study, there was no significant difference in survival rates for histopathologic T stage (pT3 vs. pT4, p=0.373). This was consistent with results reported previously.^4^^,^^13^^,^^14^ Therefore, multi-organ resection was recommended for patients with clinical T4 gastric cancer for curative resection.

Analysis of prognostic factors for T4 patients treated with multi-organ resection might help to select the optimal candidates in the future. According to our multi-variate analyses, tumor size (≤ 7cm) and curability (R0) were long-term survival factors in patients with T4 gastric cancer, who underwent multi-organ resections. Small tumor size may be associated with well-moderate differentiation, early TNM stage, and curative resection, leading to a long-time survival. Many studied identified curability (R0) as a predominant factor influencing a good prognosis for patients with T4 gastric cancer^15^, which was conformed in our study. Therefore, multi-organ resection should be performed with curative intents in patients with no other incurable factors.

Ages, resection of more than one organ and lymph node metastasis, as prognostic factors in T4 gastric cancer, had been reported in some previous literature.^16^^-^^20^ In the present study, we could not confirm the effects of these factors. The reason may be this study was a small sample size, short-term study. It was difficult to get significant difference. So a large sample size, long-term study is needed to confirm the prognostic factors in the future. In conclusion, multi-organ resection was recommended for patients with clinical T4 gastric cancer for curative resection. Each patient should be given the chance of curative resection, especially the patients with tumor diameter ≤ 7 cm.

## References

[1] Neugut AI, Hayek M, Howe G (1996). Epidemiology of gastric cancer. Semin Oncol.

[2] Siewert JR, Böttcher K, Stein HJ, Roder JD (1998). Relevant prognostic factors in gastric cancer: ten-year results of the German Gastric Cancer Study. Ann Surg.

[3] Kim DY, Joo JK, Seo KW (2006). T4 gastric carcinoma: the benefit of non-curative resection. ANZ J Surg.

[4] Carboni F, Lepiane P, Santoro R, Lorusso R, Mancini P, Sperduti I (2005). Extended multiorgan resection for T4 gastric carcinoma: 25-year experience. J Surg Oncol.

[5] Martin RC, Jaques DP, Brennan MF, Karpeh M (2002). Achieving R0 resection for locally advanced gastric cancer: is it worth the risk of multiorgan resection. J Am Coll Surg.

[6] Sobin LH, Witteking CH (2002). International union against cancer TNM classification of malignant tumours.

[7] Dicken BJ, Bigam DL, Cass C, Mackey JR, Joy AA, Hamilton SM (2005). Gastric adenocarcinoma: Review and considerations for future directions. Ann Surg.

[8] Roder DM (2002). The epidemiology of gastric cancer. Gastric Cancer.

[9] Onate-Ocana LF, Aiello-Crocifoglio V, Mondragon Sánchez R, Ruiz Molina JM, Gallardo-Rincon D (1999). Prognostic factors in 793 cases of gastric cancer in an oncologic referral center. Rev Gastroenterol Mex.

[10] Onate-Ocana LF, Becker M, Carrillo JF, Aiello-Crocifoglio V, Gallardo-Rincon D, Brom-Valladares R (2008). Selection of Best Candidates for Multiorgan Resection Among Patients With T4 Gastric Carcinoma. J Surg Oncol.

[11] Cheng CT, Tsai CY, Hsu JT (2011). Aggressive Surgical Approach for Patients with T4 Gastric Carcinoma: Promise or Myth. Ann Surg Oncol.

[12] Colen KL, Marcus SG, Newman E, Berman RS, Yee H, Hiotis SP (2004). Multiorgan resection for gastric cancer: intraoperative and computed tomography assessment of locally advanced disease is inaccurate. J Gastrointest Surg.

[13] Mita K, Ito H, Fukumoto M, Murabayashi R, Koizumi K, Hayashi T (2012). Surgical outcomes and survival after extended multiorgan resection for T4 gastric cancer. Am J Surg.

[14] Kobayashi A, Nakagohri T, Konishi M (2004). Aggressive surgical treatment for T4 gastric cancer. J Gastrointest Surg.

[15] Kim JH, Jang YJ, Park SS (2009). Surgical Outcomes and Prognostic Factors for T4 Gastric Cancers. Asian J Surg.

[16] Kunisaki C, Akiyama H, Nomura M (2006). Surgical outcomes in patients with T4 gastric carcinoma. J Am Coll Surg.

[17] Jeong O, Choi WY, Park YK (2009). Appropriate selection of patients for combined organ resection in cases of gastric carcinoma invading adjacent organs. J Surg Oncol.

[18] Ozer I, Bostanci EB, Orug T (2009). Surgical outcomes and survival after multiorgan resection for locally advanced gastric cancer. Am J Surg.

[19] Aizawa M, Gotohda N, Takahashi S, Konishi M, Kinoshita T (2011). Predictive value of baseline neutrophil/lymphocyte ratio for T4 disease in wall-penetrating gastric cancer. World J Surg.

[20] Roukos DH (2000). Current status and future perspectives in gastric cancer management. Cancer Treat Rev.

